# The Effect of Prenatal Education on Mother’s Quality of Life during First Year Postpartum among Iranian Women: A Randomized Controlled Trial

**Published:** 2013-09-18

**Authors:** Nosrat Bahrami, Masoumeh Simbar, Somayeh Bahrami

**Affiliations:** 1 Department of Midwifery, Faculty of Nursing and Midwifery, Dezful University of Medical Sciences, Dezful, Iran; 2 Department of Reproductive Health and Midwifery, Shahid Beheshti University of Medical Sciences, Tehran, Iran; 3 Department of Statistics, Dezful University of Medical Sciences, Dezful, Iran

**Keywords:** Prenatal, Quality of Life, Postpartum, Health

## Abstract

**Background:**

Antenatal educations provide information regarding pregnancy, birth, infant care and early parenthood. The purpose of this study was to determine effect of prenatal education on mother’s quality of life during first year after childbirth among Iranian women.

**Materials and Methods:**

This single-blind randomized control trial study was performed on 160 first-time pregnant women; with a singleton fetus; aged 18 to 35; without history of medical, psychological, and infertility diseases; as well as with at least eight prenatal visits during pregnancy. Participants were invited into two groups of intervention (n=80) and control (n=80). The antenatal education classes were consisted of eight sessions, and then, mother’s quality of life was evaluated during first year after childbirth. Data was analyzed using t test, chi-square, and Mann-Withney.

**Results:**

The interventional group demonstrated higher scores of quality of life domains than the control group (p<0.05). The interventional group (at one year postpartum) demonstrated significantly higher scores for quality of life in the physical health, psychological health, and environmental health domains compared to the control group. In addition, the interventional group showed a significant increase in the mean scores for the domains of physical, psychological, and environmental health from 6-8 weeks to 1 year postpartum.

**Conclusion:**

The study showed that women receiving prenatal education had higher level of happiness and satisfaction in their overall quality of life and health, respectively (Registration Number: IRCT201101115571N2).

## Introduction

Quality of life is defined as individuals' perceptions of his/her position in life, the context of culture, and value systems that they live, and also in relation to their goals, expectations, standards and concerns (1).

Quality of life has been increasingly recognized as an important outcome measure in both treatment studies and service evaluations (2). The postpartum period is an important time for a mother, her newborn and family. Women problems may increase during the mentioned period (3, 4), therefore, these changes may impact on mother’s wellbeing (5). Additionally, women reports considerable limitations in their abilities to function at work and at home even up to 6 months postpartum (6, 7). Antenatal education provides information regarding pregnancy, birth process, infant care, and confidence in taking of the responsibilities of parenthood (8), which is admitted as a positive approach to prepare the women for child birth (9). Antenatal education reveals numerous effects on women’s quality of life at the postpartum (10). Some studies have found significant beneficial effects of antenatal education and/or support programs on the quality of birth, postpartum experiences and the satisfaction of the parents (11, 12). Variety of medical, psychological, social and obstetric factors could have impact on the quality of life after the postpartum. Mothers’ negative perception of their own health can have a negative impact on their infant care behaviors. The health of women after childbirth may affect their children’s health (13).

Assessing quality of life in this period will allow a woman to make a self-evaluation of her own postnatal situation, and will also assist health care providers for further promotion of women and infants’ health. Akyn et al. (14) indicated that quality of life of mothers who were able to receive emotional support from their families and spouses showed much more positive score in quality of life compared to mothers who did not receive this support. (14). But Artieta-Pinedo et al. (15) showed that antenatal education is not associated with any benefits for childbirth. The most recent meta-analysis has concluded that there is insufficient evidence to determine the effects of antenatal education on psychological, physical and social adjustment (9). Therefore, the purpose of the present study was to determine the effect of prenatal education on mother’s quality of life during their first year after childbirth among Iranian women.

## Materials and Methods

This single-blind randomized control trial was performed between June 2010 and December 2011. The 160 pregnant women were randomly assigned into two groups using a random sampling method. Interventional group included 80 participants (received prenatal education classes), while control group included 80 participants (not received prenatal education classes). Participants are referred to Health and Treatment Center of number one, Dezful, Iran. All participants (intervention group and control group) were enrolled in the routine prenatal care classes. First-time pregnant women with a singleton fetus were invited to participate in the study if they met the following criteria: i. aged 18 to 35, ii. between 24 and 28 weeks’ gestation, iii. without history of medical, psychological, and infertility diseases, iv. with at least eight prenatal visits during pregnancy, whilst two visits occurred before the 20th week of pregnancy, v. reading and writing in Farsi (Persian), and vi. giving written informed consent. Women were excluded if they met the following exclusion criteria: i. neonate with congenital anomalies, ii. neonatal death, iii. complicated pregnancy, iv. drug consumption, v. birth weight <2500 gram, vi. postpartum depression, vii. family difficult during six month after child birth, and viii. failure to attend 2 sessions of prenatal education classes. The antenatal education classes were eight daytime sessions for 90 minutes in a period of 3 months (2-4 sessions per month at 9-10:30 am.), located at the Health and Treatment Center of number one, Dezful, Iran. Classes included 8-10 mothers. 

The antenatal education class, presented by an experienced midwife, consisted of the following programs: anatomical, physiological, and psychological changes during pregnancy; a proper diet during pregnancy; stages of natural labor; care of the mother and newborn following delivery; breastfeeding; and family planning. Training was carried out theoretically using educational and audiovisual devices, such as television and computer. Proper position during labor and delivery, the correct way of breathing during pregnancy, relaxation, and neuromuscular exercises were introduced, and also, were practically performed. A counseling session was also held to answer all questions of participated mothers. The Ethics Committee of Dezful University of Medical Sciences approved the study protocol.

llection consisted of a demographic questionnaire, as well as the Iranian interview-administered version of the World Health Organization’s Quality of Life Questionnaire (WHOQOL-BREF). The WHOQOL-BREF is a 26-item instrument including the following four domains: physical health (7 items), psychological health (6 items), social relationships (3 items), and environmental health (8 items), as well as two overall quality of life and general health items. All scores are transformed to reflect 4 to 20 for each domain with higher scores corresponding to a better quality of life. There is no overall score for the WHOQOL-BREF (16). Content validity and test-retest were performed to assess validity and reliability of the questionnaire (16, 17). The questionnaires completed between 24^th^ and 28th weeks’ gestation (for your information: 1^st^, 2^nd^, 3^rd^, 4^th^, 5^th^, etc), 6-8 weeks after childbirth, as well as 1 year postpartum. Data was analyzed by SPSS 15 (Chicago, IL, USA) using t test, Chi-square, and Mann-Withney. Statistical significance was considered to be p<0.05.

## Results

One hundred sixty women with mean age of 24.2 ± 4.05 years (mean ± SD) participated in the study. Their demographic and fertility characteristics were compared and summarized in table 1. As shown in table 1, no significant differences between the groups were found regarding the possible confounding variables. 

**Table 1:** The comparison of individual and fertility characteristics of mothers in the interventional and the control groups in the Dezful Health Center


Group characteristics	Interventional group n=80	Control group n=80	P value^a^

**Individual**			
Age (Y): mean ± SD	24.6 ± 3.9	23.8 ± 4.2	NS
BMI (kg/m2): mean ± SD	26.8 ± 4.3	26.1 ± 3.6	NS
**Occupation N(%)**			
Housewife	74 (92.5)	77 (96.2)	
Employed	6 (7.5)	3 (3.8)	NS
**Woman’s educational years: N (%)**			
< 12years	58 (72.5)	52 (65)	
> 12years	22 (27.5)	28 (35)	NS
**Spouse’s educational years: N (%)**			
< 12years	43 (53.8)	41(51.2)	
> 12years	37 (46.2)	39 (48.8)	NS
**Social-economical status: N (%)**			
Good	14 (17.5)	17 (21.2)	
Intermediate	66 (82.5)	63 (78.8)	NS
**Fertility**			
**Mode of delivery: N (%)**			
Vaginal delivery	52 (65)	48 (60)	
Cesarean section	28 (35)	32 (40)	NS
**Gender of neonate: N (%)**			
Female	37 (46.2)	38 (47.5)	
Male	43 (53.8)	42 (52.5)	NS


a; t test and Chi- Square test were used and NS; Non significant.

Mothers (at 6-8 weeks postpartum in the interventional group) rated their overall quality of life as follows: i. very good (17%), ii. good (58%), iii. neither good nor bad (20%), iv. bad (4%), and v. very bad (1%). Participants rated level of satisfaction of their health as follows: i. very satisfied (16%), ii. satisfied (52%), iii. neither satisfied nor dissatisfied (24%), iv. dissatisfied (6%), and v. very dissatisfied (2%). But, control group showed a decrease in percentages of quality of life and health levels compared to the interventional group. Table 2 presents the mean and standard deviation for domains of mother’s quality of life in the interventional and the control groups (at 6-8 weeks postpartum). As shown in table 2, the interventional group demonstrates higher scores for different domains of quality of life (p<0.05) compared to those of the control group.

The differences were statistically significant for domains of physical health, psychological health, social relationships and environmental health between two groups (p<0.05). The domain of physical health showed the highest score, while the domain of environment health showed the lowest score among the domains of WHOQOL-BREF (at 6-8 weeks postpartum). The results revealed significant differences among the mean scores for domains of the physical health, psychological health, and environmental health between two groups (at 1 year postpartum). In this study, the interventional group (at 1 year postpartum) was more likely to demonstrate higher scores for different domains of quality of life, including physical health, psychological health, and environmental health domains in comparison to the control group (p<0.05). In the current study, the interventional group had higher scores on social relationships, but the difference was not statistically significant. Table 3 depicts the four mean scores belonging to the domains of the quality of life (at 1 year postpartum). In addition, the interventional group showed a significant increase in the mean scores of three domains belonging to physical health, psychological health, and environmental health, from 6-8 weeks postpartum to 1 year postpartum (p<0.05). But, the control group revealed a significant increase in the mean scores of two domains belonging to physical health and environmental health, from 6-8 weeks postpartum to 1 year postpartum (p<0.05).

**Table 2:** The comparison of mean and standard deviation (SD) per domains of mother’s quality of life in the interventional and the control groups at 6-8 weeks postpartum


Group characteristics	Interventional group n=80	Control group n=80	P value^b^

**Physical health: mean ± SD^a^**	15.1 ± 1.8	12.9 ± 2.3	0.000
**Psychological health: mean ± SD^a^**	13.9 ± 2.3	13.2 ± 1.6	0.017
**Social relationships: mean ± SD^a^**	14.3 ± 4.3	13.8 ± 3.6	0.01
**Environment health: mean ± SD^a^**	13.1 ± 4.3	12.2 ± 3.6	0.000


a; The higher score represents a better condition (scores range from 4 to 20) and b; t test was used.

**Table 3:** Mean (± SD) of mother’s quality of life in the interventional and the control groups at 1 year postpartum


Group characteristics	Interventional group n=80	Control group n=80	P value^b^

**Physical health: mean ± SD^a^**	15.7 ± 1.7	14.4 ± 2.8	0.000
**Psychological health: mean ± SD^a^**	14.8 ± 2.6	13.5 ± 1.6	0.002
**Social relationships: mean ± SD^a^**	14.5 ± 3.1	14.2 ± 3.1	0.17
**Environment health: mean ± SD^a^**	13.9 ± 2.4	13.1 ± 2.9	0.01


a; The higher score represents a better condition (scores range from 4 to 20) and b; t test was used.

## Discussion

To the best of our knowledge, this is the first large randomized controlled interventional trial of antenatal group education that includes long-term follow up of the mother’s quality of life between 6-8 weeks postpartum and 1 year postpartum.

The majority of participants in the interventional group reported that their overall quality of life and health were good and satisfactory, respectively, according to the WHOQOL-BREF questionnaire. In the study by Zubaran et al. (17), they have reported the women in the interventional group have higher level of happiness and satisfaction of their overall quality of life and health compared to the control group, which was similar to our obtained result. Postpartum women should cope with body changes and new responsibilities of motherhood (14). Hill and Aldag (18) indicated that happy mothers reported a higher level of satisfaction in their relationship with their spouses; in addition, there was a significant increase in the level of health and general functioning during the study. But Nagpal et al. (19) showed that the scores of overall quality of life were lower than that in our study. Therefore, according to our obtained results, prenatal educations are one of the reasons for higher level of happiness and satisfaction in the overall quality of life and health, respectively, among the participants. Lack of awareness can cause fear and anxiety during pregnancy and birth, then these may induce complications during delivery and postpartum, and also, these may be one of the reasons that women ask for medical interventions (20, 21). 

In the interventional group, the high mean scores for domains of the quality of life at 6-8 weeks postpartum indicated higher level of physical, psychological, social relationships and environmental health compared to the control group. Results from the study by Zubaran et al. (17) indicated that a sample of postpartum mothers showed favorable scores for domain of the quality of life. However, other researches indicated that women showed slightly satisfaction with their lives during the postpartum period (3). But, IP et al. reported that the educational intervention is effective in promoting pregnant women’s self-efficacy for childbirth, while reduces their perceived pain and anxiety in the first two stages of labor. Relief of pain and anxiety is an important issue for both women and health-care professionals. The self-efficacy education should be more for mothers during pregnancy (9). In a study showed that taking severe exercise after delivery is more associated with the social and mental wellbeing (22). In addition, in the interventional group, the high mean scores for domains of the quality of life is the result of having the knowledge of physiologic and phsychological changes during pregnancy and the postpartum period, including factors associated with recovering from the birthing process and caring of newborns. Further support for this conclusion comes from the study by Hueston and Kasik-Miller (23) in which suggested that pregnancy was the cause of the changes. In a study by Zubaran et al. (17) conducted in order to assess the postpartum period, participated mothers reported a sense of improvement and high self-esteem, as well as good or improved relationships with their family members.

In our study, the interventional group (at 1 year postpartum) demonstrated higher scores for domains of quality of life (QOL), like physical health, psychological health, and environmental health compared with the control group. Quality of life of mothers receiving support from their families and spouses was much more positive score compared to whom did not receive this support. In many studies, a relationship between emotional support and the health level of the mother has been detected (24-26). Other studies showed that recovery of physical and social functioning after delivery requires longer than 6 weeks (27).

The significant increase in domains at 1 year postpartum compared with 6-8 weeks postpartum may indicate additional demands by pregnant women to receive this prenatal education. In a study by Hill and Aldag they assessed the quality of life on 184 mothers, and revealed a significant increase in terms of their health and mental functioning during the study. From weeks 1-3, the mean scores for domains of the health and functioning have shown significant increase for mothers of intervention group. Mothers can be informed that their satisfaction with their health and mental functioning, as well as overall QOL will likely improve with time. The overall mean QOL scores for mothers was higher at the week 3 compared to the first week postpartum. This finding suggests that this sample of mothers was more satisfied with their lives in general as time passed (18). The results showed that mothers in the interventional group have higher levels for domains of quality of life.

## Conclusion

Our findings suggest that the mother’s knowledge could potentially reduce their levels of anxiety and stress in pregnant women, which in turn, may improve maternal health and quality of life during first year postpartum. Further research on the effect of prenatal education on mother’s quality of life during breastfeeding or up to 5 years after childbirth is required in order to enhance our existing knowledge. 
